# How Membrane Contact Sites Shape the Phagophore

**DOI:** 10.1177/25152564231162495

**Published:** 2023-05-09

**Authors:** Cristina Capitanio, Anna Bieber, Florian Wilfling

**Affiliations:** 1 Department of Molecular Machines and Signaling, 28311 Max Planck Institute of Biochemistry, Martinsried, Germany; 2 Aligning Science Across Parkinson's (ASAP) Collaborative Research Network, Chevy Chase, MD, USA; 3 Mechanisms of Cellular Quality Control, 28273 Max Planck Institute of Biophysics, Frankfurt a. M., Germany

**Keywords:** membrane contact sites, autophagy, ER, vacuole, lipid transfer

## Abstract

During macroautophagy, phagophores establish multiple membrane contact sites (MCSs) with other organelles that are pivotal for proper phagophore assembly and growth. In *S. cerevisiae*, phagophore contacts have been observed with the vacuole, the ER, and lipid droplets. In situ imaging studies have greatly advanced our understanding of the structure and function of these sites. Here, we discuss how in situ structural methods like cryo-CLEM can give unprecedented insights into MCSs, and how they help to elucidate the structural arrangements of MCSs within cells. We further summarize the current knowledge of the contact sites in autophagy, focusing on autophagosome biogenesis in the model organism *S. cerevisiae*.

## Introduction

**Figure 1.**
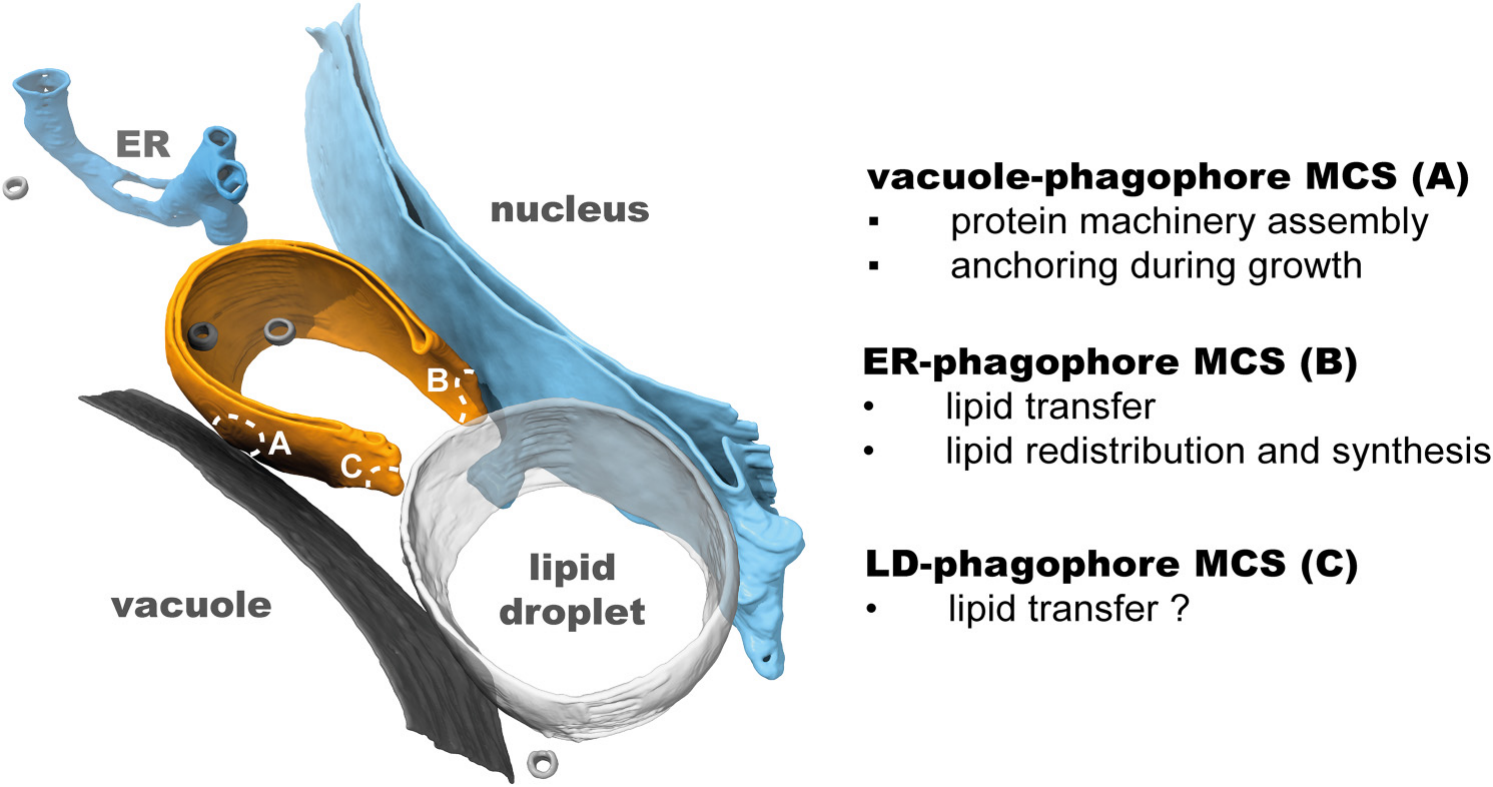
Membrane contact sites (MCSs) contribute to phagophore formation and growth. The double membrane phagophore (orange) establishes multiple contact sites during growth. It is anchored to the vacuole at its side or back (A), whereas its rim establishes contact with the ER, including tubular ER (see Figure 3) and the nuclear membrane (B). In addition, it can also directly interact with lipid droplets (C). In this example, all three interorganelle MCSs are captured in situ in *S. cerevisiae* by correlative cryo-ET and can be appreciated in the 3D rendering of the segmented membranes (Bieber et al., 2022; EMD-15548).

Macroautophagy (hereafter referred to as autophagy) is a conserved pathway that allows eukaryotic cells to degrade intracellular components such as invading pathogens, protein aggregates, or damaged organelles to maintain cellular homeostasis both under physiological and stress conditions. The hallmark of autophagy is the de novo synthesis of an autophagosome, a double-membrane organelle that sequesters cargo and fuses with a lytic compartment for degradation. Autophagosome formation is fast—it usually gets completed in 10 minutes (Geng et al., 2008; Schütter et al., 2020; Tsuboyama et al., 2016). To achieve this, autophagy critically depends not only on a conserved protein machinery, but also on other organelles that make contact with the growing structure and provide an assembly platform and lipids for expansion (Figure 1). Studying the membrane contact sites (MCSs) in autophagy is thus crucial for a holistic understanding of this important degradation pathway. In the following, we will review the current knowledge on phagophore-organelle MCSs and discuss how methods like cryo-CLEM and cryo-ET are helping to advance our understanding of these important sites in the cell.

## Membrane Contact Sites

To date, membrane contact sites (MCSs) have been described for nearly all possible pairs of common eukaryotic organelles (Gatta and Levine, 2017). As per definition, MCSs are areas of close proximity between organelle membranes that do not result in fusion and fulfill specific functions, such as organelle remodeling or transport of lipids or ions (Scorrano et al., 2019). MCSs are constituted of protein and lipid components, which are specific for the organelles involved and the function of the respective contact site. Proteins at contact sites include tethers with purely structural roles, the machinery necessary to perform specific functions, and regulatory proteins. The protein population both defines and is defined by the specific local shape and lipid composition of the two opposing membranes. While biochemistry and in vitro experiments are indispensable for a detailed molecular analysis of known MCS components, only comprehensive studies of the organellar contact network directly within cells can reveal previously unknown MCSs and elucidate their structures and dynamics. Fortunately, a vast toolbox of microscopy methods is now available to characterize MCSs in situ. The most well-established methods include different fluorescence and electron microscopy techniques and their combination: correlative light and electron microscopy (CLEM).

## The Role of CLEM and Cryo-ET in the Study of Contact Sites

Over the past decades, fluorescence microscopy has played a key role in discovering and studying the dynamics of many contact sites (Scorrano et al., 2019). The use of organelle-specific fluorescent labels has helped to understand the extent of interconnectivity between organelles by mapping the frequency of contact sites in mammalian cells (Valm et al., 2017). In addition, proximity assays such as fluorescence resonance energy transfer (FRET) measurements and split fluorescence systems have been instrumental to characterize the microenvironment of MCSs (Csordás et al., 2010; Scorrano et al., 2019; Shai et al., 2018; Venditti et al., 2019) and to study the dynamics of such contacts under different stimuli (Yang et al., 2018).

While fluorescence microscopy is well-suited for characterizing the interactions and dynamics of specific proteins even in live cells, it is limited in resolution and leaves all unlabeled structures in the dark. In contrast, electron microscopy methods reveal the entire local cellular environment at a higher resolution, making use of the inherent contrast of the biological structures, sometimes enhanced with nonspecific stainings (Kuchenbrod et al., 2021). However, given the small mean free path of electrons, imaging needs to be done in vacuum, necessitating sample fixation by chemical methods or vitrification. Serial block face imaging of samples with focused ion beam–scanning electron microscopy (FIB-SEM) yields whole-cell 3D maps at intermediate resolutions (3–10 nm resolution (Xu et al., 2017)) and was used, for example, to characterize the ER connectome in different areas of neurons (Wu et al., 2017). Even higher resolutions can be obtained with transmission electron microscopy (TEM), but for most cells, this requires prior thinning to make the specimen electron transparent. For room temperature TEM, chemically or cryo-fixed samples are embedded in resin and cut into thin sections which can then be used for 2D imaging or 3D tomography. The advantages of the method include the relatively high throughput and the possibility to reconstruct larger 3D volumes by combining data from adjacent sections, showcased in a study providing detailed maps of the ER structure and its contacts in *S. cerevisiae* (West et al., 2011).

To resolve high-resolution details and even protein structures in situ without introducing fixation artifacts (Dubochet and Sartori Blanc, 2001), cryo-electron tomography (cryo-ET) is the prevalent method (Collado and Fernández-Busnadiego, 2017; Turk and Baumeister, 2020). After vitrification, cryo-focused ion beam (FIB) milling is usually used to remove material from the top and bottom of the cell, leaving a ∼150-nm-thin lamella suitable for tilt series acquisition (Schaffer et al., 2017). Developments in FIB milling automation (Klumpe et al., 2021; Kuba et al., 2021; Tacke et al., 2021), lift-out for thick samples (Klumpe et al., 2022; Schaffer et al., 2019), and plasma FIB (Berger et al., 2023; Rigort et al., 2022) are dramatically increasing the throughput and scope of samples, thereby promising to establish this technique as a routine method in the near future.

Cryo-ET has been a pivotal tool in the investigation of the fine structures of several MCSs including ER-PM contacts in *S. cerevisiae* (Collado et al., 2019; Hoffmann et al., 2019). A precise analysis of the local membrane shape, including distance and curvature maps, can be used to define consensus structures and reveal the heterogeneity of these areas, with direct implications for contact site function (Collado et al., 2019). Secondly, cryo-ET can in theory resolve structures of proteins at the contact site by subtomogram averaging if they are sufficiently large and abundant (Castaño-Díez and Zanetti, 2019). However, for most contact sites, this is challenging given the heterogeneous protein populations, conformational flexibility, and low copy numbers of most MCS-associated proteins (Cai et al., 2022; Hoffmann et al., 2019). Still, in the example of an in situ cryo-ET study of the ER-mitochondria encounter structure (ERMES), subtomogram averaging provided a sufficiently resolved map that, combined with in vitro data and computational approaches, yields new insights into protein arrangements with important functional implications (Wozny et al., 2022).

While fluorescence and electron microscopy are powerful methods, correlative light and electron microscopy (CLEM) combines high resolution with fluorescence targeting of specific cellular structures. Room temperature and cryo-CLEM have been described in detail in a previous review (Ganeva and Kukulski, 2020). Fluorescence information can be used not only to guide TEM data acquisition and interpretation but also for sample preparation by cryo-FIB milling, where 3D correlative milling helps to ensure that the structure of interest is retained in the lamella (Arnold et al., 2016; Bieber et al., 2021). Depending on the MCSs and the organelles forming them, fluorescence labeling can either target one or both organelles (Bieber et al., 2022) or specific contact site proteins (Wozny et al., 2022). Many of the aforementioned methods have been instrumental in reaching our current understanding of the MCSs in autophagosome biogenesis. In the following, we will give a brief overview of autophagy, followed by a detailed discussion of the individual MCSs involved in this process.

## Autophagosome Biogenesis

Autophagy and its proteins are highly conserved from yeast, where it was characterized originally, to higher eukaryotes such as plants and mammals (Mizushima et al., 2011; Yin et al., 2016). Bulk autophagy describes the nonselective uptake and degradation of cytosolic contents to recycle nutrients as a cellular response to starvation. In addition, autophagy can also target specific cargo through selective autophagy receptors, which link the autophagy machinery to the cargo, promoting its selective sequestration into the autophagosome (Gatica et al., 2018; Lamark and Johansen, 2021). De novo biogenesis of autophagosomes is characterized by the hierarchical assembly of core autophagy machinery proteins to the expanding cup-shaped autophagosome precursor membrane, called isolation membrane or phagophore. This process has been well summarized in various comprehensive reviews (Hollenstein and Kraft, 2020; Nakatogawa, 2020). Here, we will briefly summarize the steps leading to autophagosome formation in *S. cerevisiae*, the currently best-characterized model system.

Autophagosome biogenesis in *S. cerevisiae* takes place on the vacuole at the phagophore assembly site (PAS). Under bulk autophagy conditions, when no defined cargo is present, PAS initiation is dependent on the oligomerization and the phase separation of the Atg1 kinase complex, which induces clustering and activation of the kinase Atg1 (Fujioka et al., 2020; Kamber et al., 2015; Torggler et al., 2016; Yamamoto et al., 2016). Tethering of the PAS to the vacuole is mediated by the interaction of the vacuolar protein Vac8 with several autophagy machinery proteins, including Atg13, a member of the Atg1 kinase complex, and Atg11, which also interacts with cargo via selective autophagy receptors (Hollenstein et al., 2019; Scott et al., 2000; Shintani et al., 2002). The Atg1 complex and Atg11 recruit small vesicles with the transmembrane protein Atg9 to the PAS, which are thought to fuse to form the initial concave membrane disk (He et al., 2006; Mari et al., 2010; Sekito et al., 2009; Suzuki et al., 2015; Yamamoto et al., 2012). Next, the phosphatidylinositol-3 kinase (PI3K) complex I generates phosphatidylinositol-3-phosphate (PI3P) in the phagophore membrane, which in turn leads to the recruitment of the Atg2/Atg18 complex (Kihara et al., 2001; Krick et al., 2006; Obara et al., 2008a, 2008b). This complex acts as a bridge between the phagophore rim and the ER and is thought to fuel phagophore expansion by channeling lipids from the ER through Atg2 into the phagophore membrane (Maeda et al., 2019; Osawa et al., 2019; Valverde et al., 2019). In addition to lipid transfer from the ER and fusion of Atg9 vesicles, COPII vesicles have also been shown to fuse with and contribute to the growing phagophore (Shima et al., 2019). The highly curved rim is thought to induce the characteristic cup shape of the growing phagophore, since this shape reduces the rim circumference and thus the bending energy of the structure (Knorr et al., 2012; Sakai et al., 2020). The growing membrane is further modified by conjugation of the ubiquitin-like protein Atg8 (or its homologs LC3/GABARAP in higher eukaryotes) to the lipid phosphatidylethanolamine (PE). Among other functions, Atg8 interacts with selective autophagy receptors to mediate the enwrapping of specific cargo by the growing phagophore (Johansen and Lamark, 2020). Phagophore closure is a membrane scission process and was suggested to depend on the ESCRT (endosomal sorting complex required for transport) machinery (Takahashi et al., 2018; Zhou et al., 2019). Maturation of the closed autophagosome involves the removal of Atg8 and PI3P from its outer membrane, which is followed by its SNARE-mediated fusion with the vacuole (Reggiori and Ungermann, 2017). As a result, a single membrane vesicle containing the cargo, called the autophagic body, is released into the vacuole, where its degradation is mediated by the lipase Atg15 and further vacuolar hydrolases (Epple et al., 2001).

The two MCSs of the phagophore described so far—with the vacuole and the ER—are also its two best-understood contact sites. In addition, other organelles such as lipid droplets and mitochondria (in mammalian cells) have been observed to form MCSs with phagophores (Figure 1) (Bieber et al., 2022; Dupont et al., 2014; Hailey et al., 2010; Hamasaki et al., 2013; Shpilka et al., 2015). While these interactions may be important in specific cases, they have been observed less consistently in either mammalian or yeast autophagy (Graef et al., 2013; Takahashi et al., 2022), arguing for a nonessential or cargo-specific function during autophagosome biogenesis. Moreover, there are nonphagophore MCSs with potential roles in autophagy, such as the ER–mitochondria and ER–plasma membrane MCSs that regulate local PI3P synthesis (Böckler and Westermann, 2014; Hamasaki et al., 2013; Nascimbeni et al., 2017). The role of these nonphagophore MCSs has been reviewed elsewhere (Kohler et al., 2020; Zwilling and Reggiori, 2022). Here, we will focus in detail on the three best-characterized MCSs of the phagophore, with the vacuole, ER, and LDs.

## The Vacuole–Phagophore Contact Site

### The PAS is Anchored to the Vacuole Through Vac8

**Figure 2.**
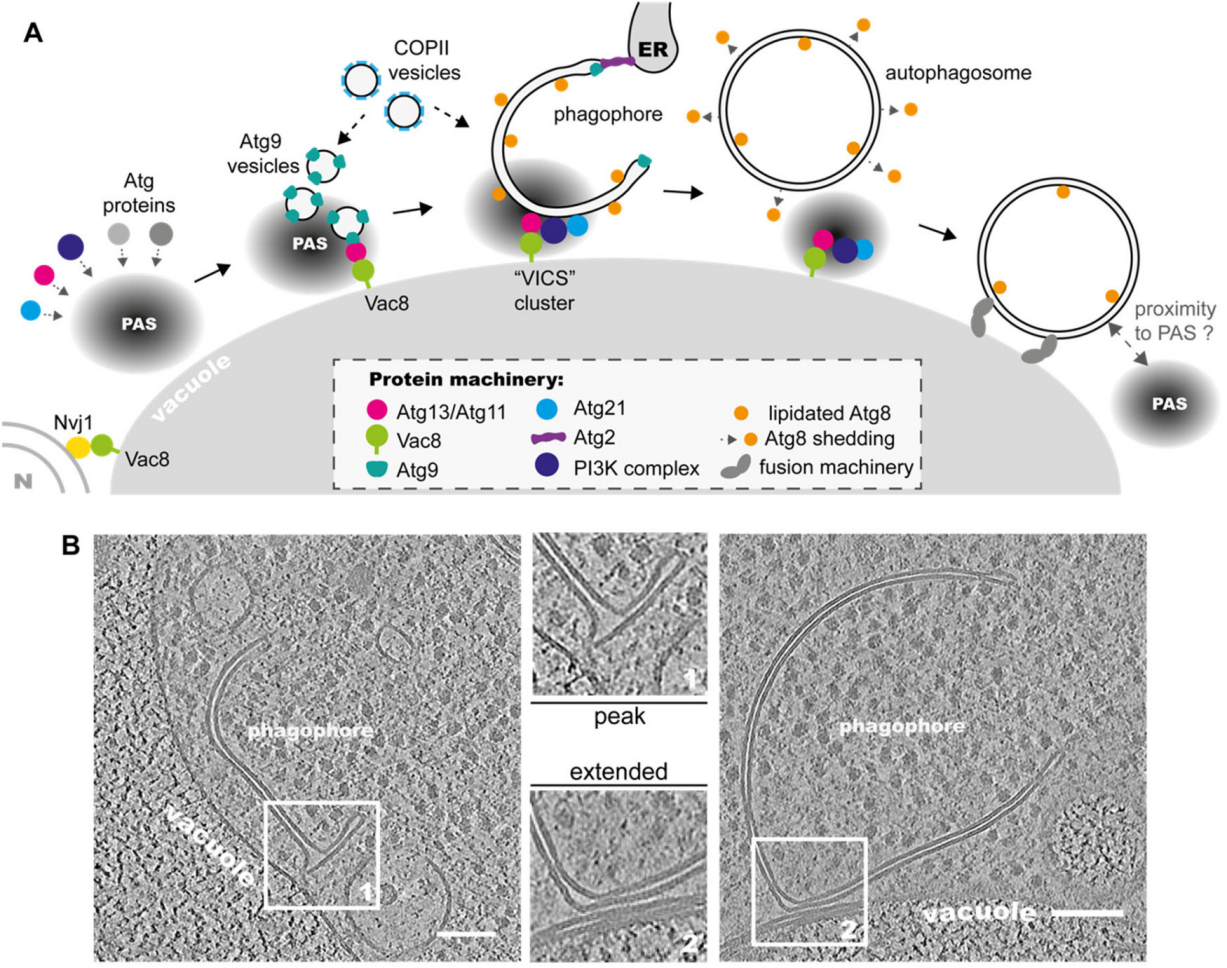
The yeast vacuole acts as a platform for PAS assembly and phagophore anchoring. A. The vacuolar protein Vac8 mediates the contact with the Phagophore Assembly Site (PAS) machinery via its interaction with Atg13 or Atg11. Vac8 is also involved in the formation of the vacuole MCS with the nucleus (N). Atg9 vesicles are docked at the PAS through the Atg1 complex and/or Atg11 and represent the nucleation point for phagophore formation. COPII vesicles also contribute to phagophore growth. Specific autophagy proteins gather at the phagophore–vacuole MCS to form the “VICS cluster”. At this stage, Atg8 is conjugated to the phagophore membrane, while Atg9 localizes to the rim, where the ER–phagophore MCS is established. During maturation, Atg8 is removed from the outer membrane and the autophagosome is thought to lose contact with the vacuole. Finally, the fusion machinery mediates tethering to the vacuole and membrane fusion, leading to subsequent degradation of the autophagosome. B. The strong tethering of the phagophore to the vacuole at the MCS is shown in tomogram slices of *in situ* correlative cryo-ET. Two different morphologies of the contact site are observed: peak (1) or extended (2). Scale bars 100 nm (Adapted from Bieber et al., 2022; left tomogram: EMD-15549).

The yeast vacuole acts as a platform for the biogenesis of the phagophore: it is both an assembly point for the autophagic machinery and an anchoring site of the maturing phagophore (Figure 2A). The first step in establishing this contact is the formation of the PAS, which was first described as a perivacuolar punctate structure in which several autophagy proteins colocalize (Suzuki et al., 2001). In a seminal study published in 2019, Hollenstein et al. showed that the PAS is anchored to the vacuole through Vac8 (Hollenstein et al., 2019), a protein known for its role in vacuole inheritance and fusion, nucleus–vacuole junctions, and the cytoplasm-to-vacuole (Cvt) pathway (Pan and Goldfarb, 1998; Pan et al., 2000; Wang et al., 1998). Vac8 has acylation sites at the N-terminus, which are important for its membrane localization and function in autophagy (Boutouja et al., 2019; Gatica et al., 2021; Hollenstein et al., 2019; Wang et al., 1998). It consists of 12 armadillo (ARM) repeats (Jeong et al., 2017; Wang et al., 1998) which mediate its dimerization (Park et al., 2020) and binding to Nvj1 (Jeong et al., 2017) but also the recruitment of the C-terminus of the early autophagy protein Atg13 (Park et al., 2020; Scott et al., 2000). Interaction with Atg13 was shown to be essential for stable anchoring of the PAS to the vacuole during bulk autophagy (Hollenstein et al., 2019). Vac8 was also shown to mediate PAS-vacuole tethering during selective autophagy via its direct and independent interactions with the PI3KC1—likely via Vps34 and/or Atg6 — and Atg11 (Hollenstein et al., 2021; Lei et al., 2021).

### The Phagophore Membrane Grows While Tethered to the Vacuole

Following the assembly of the PAS machinery at the vacuole, the phagophore is thought to be generated by the fusion of Atg9 vesicles, which are recruited by Atg13 and/or Atg11 and are thus likely to be placed in close contact with the vacuole from the beginning (He et al., 2006; Suzuki et al., 2015; Yamamoto et al., 2012). How does the relationship between the vacuole, the PAS machinery, and the phagophore evolve during membrane elongation? To analyze the differences in the localization of the autophagy machinery proteins on phagophores during selective autophagy with diffraction-limited fluorescence microscopy, Suzuki, Ohsumi and colleagues overexpressed the Cvt cargo protein prApe1 that leads to the formation of giant Ape1 complexes. Phagophores appeared to be unable to close and mature around the giant Ape1 cargo and instead stopped growing after reaching a limited size (Suzuki et al., 2013). Analyzing the localization patterns of different autophagy proteins relative to the phagophore, one of three distinct clusters was found to localize to the vacuole–phagophore contact site, termed “VICS” (“vacuole-isolation membrane contact site”). This group contained mostly early machinery proteins, including subunits of PI3KC1, Atg13, and Atg17. Interestingly, even though Atg1 forms a complex with Atg13 and Atg17, it is not confined exclusively to the VICS as GFP-Atg1 was found all over the stalled phagophore (Suzuki et al., 2013). Apart from the VICS-localized proteins found in the first study using giant Ape1, another study using the same technique showed that Vac8 and Atg21 likewise localize to the VICS (Munzel et al., 2021). Atg21 binds PI3P on the phagophore membrane and promotes local lipidation of Atg8 by recruiting both Atg8 and its E3-like complex (Juris et al., 2015). While it makes sense that it binds to PI3P locally produced by PI3KC1, this is unlikely to be the only mechanism confining Atg21 to the contact site as PI3P redistributes throughout the phagophore and is also present on other cellular membranes (Cheng et al., 2014; Munzel et al., 2021). Of note, Vph1, a subunit of the vacuolar V-ATPase, was excluded from the VICS, whereas the small phosphatase Pho8 remained distributed evenly on the vacuole (Munzel et al., 2021). This exclusion of large proteins like Vph1 is reminiscent of other contact sites like the nuclear–vacuolar junction and points to a distinct molecular composition of the vacuole-phagophore MCS.

Which part of the phagophore makes contact with the vacuole? Fluorescence microscopy using giant Ape1 cargo suggested that the VICS localizes close to the phagophore rim (Munzel et al., 2021; Suzuki et al., 2013). In contrast, fluorescence imaging of phagophores without Ape1 overexpression but with deletion of the Cvt cargo receptor Atg19 reported by Graef and colleagues indicated that Atg13 and PI3KC1 (represented by Atg14) do not localize specifically to the rim. Moreover, in the same study, it was shown that phagophores often span between the vacuole and ER with the rim towards the latter (Graef et al., 2013). In line with this finding, cryo-CLEM experiments revealed that the large majority of vacuole contacts were not localized to the phagophore rim but rather to the side or back of the phagophores, leaving the opening to face the cytosol and interact with the ER or nucleus (Bieber et al., 2022). Importantly, phagophores were often deformed at the contact site, pointing to direct tethering between the two membranes. Most deformations fit into one of the following two categories: (1) a peak of the outer phagophore membrane towards the vacuole or (2) an extended contact between the two closely associated membranes, often accompanied by local flattening (Figure 2B). Deformations of both categories were highly heterogeneous with large variations in contact area, minimum distance, peak shapes, and curvature (Bieber et al., 2022). This range of morphologies can be explained by a highly variable number of local tethering complexes, potentially involving Vac8 clusters of different sizes, combined with random relative movements of the two organelles. Moreover, the peak-like membrane deformations observed by cryo-ET indicate that tethering between the organelles must be strong enough to withstand the forces needed to induce such high curvature.

### Closing a Chapter: the Phagophore–Vacuole Contact Site After Autophagosome Closure

How long is the phagophore–vacuole contact site maintained, and is it functionally related to the autophagosome–vacuole fusion site? Maturation of closed autophagosomes includes the shedding of Atg8 and PI3P (Cebollero et al., 2012; Cheng et al., 2014; Kirisako et al., 2000; Nair et al., 2012; Nakatogawa et al., 2012; Yu et al., 2012), and it is conceivable that the molecular changes are accompanied by changes in membrane morphology and contact site stability. With cryo-ET, many already elongated phagophores were observed to be tethered to the vacuole, while closed autophagosomes showed very rarely clear vacuole contacts. However, often fairly intact autophagic bodies were observed immediately next to phagophores, suggesting that phagophore formation and fusion are locally coupled (Bieber et al., 2022). Interestingly, deletion of Vac8 reduces the fusion efficiency of autophagosomes both in cells and in vitro (Hollenstein et al., 2019). There is currently no evidence that the fusion machinery localizes specifically to the phagophore–vacuole contact site. While the R-SNARE protein Ykt6 is already recruited to the early phagophore, imaging experiments with giant Ape1 complexes showed that it distributes uniformly on the phagophore membrane with no preference for the VICS (Gao et al., 2020). However, Ykt6 on growing phagophores is in a fusion incompetent state since its phosphorylation by Atg1 inhibits its interaction with the Q-SNAREs Vam3 and Vti1 on the vacuole, preventing premature and nonproductive fusion events (Barz et al., 2020; Gao et al., 2020). How dephosphorylation of Ykt6 is regulated upon autophagosome closure and if this affects its suborganellar localization remains to be determined.

## Phagophore–ER Contact Site

### The Phagophore is Anchored at its Rim to the ER

**Figure 3.**
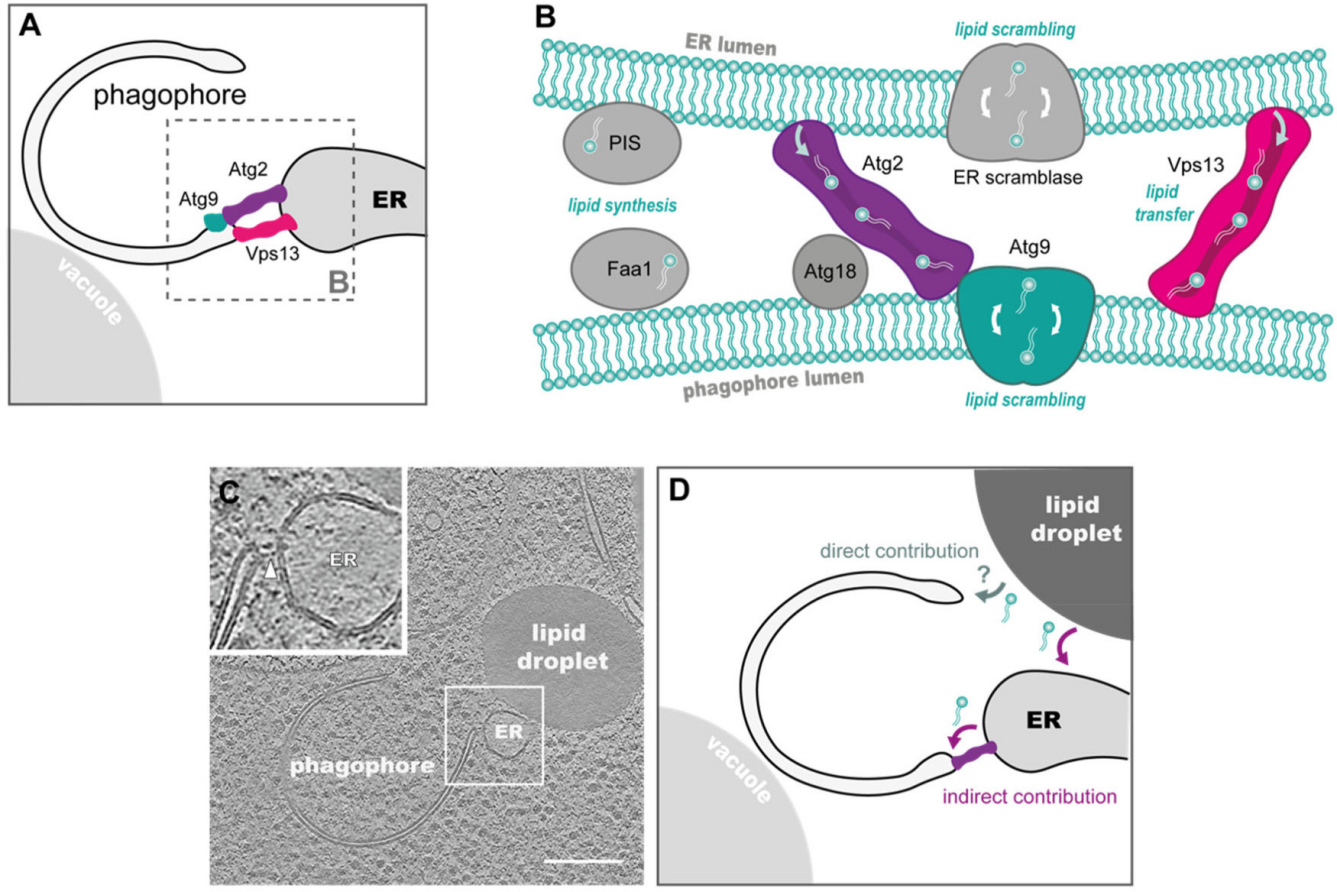
The roles of phagophore–ER and phagophore–lipid droplet MCSs in phagophore membrane expansion. A. The phagophore–ER contact is established via the phagophore rim. Proteins at this MCS include the lipid shuttling proteins Atg2 and Vps13 and the transmembrane scramblase Atg9. B. Direct lipid transfer is mediated by Atg2 and Vps13 at the ER–phagophore MCS. Atg2 interacts with Atg18 and Atg9 to contact the phagophore membrane. The lipids are most likely inserted in the outer leaflet of the phagophore membrane and then shuffled between leaflets by Atg9. Further scramblases are thought to have the same role on the ER side and have been identified in mammalian cells, but not yet in yeast. Moreover, the presence of lipid biosynthesis enzymes on both sides of the MCS, e.g., Faa1 in yeast or phosphatidylinositol synthase (PIS) in mammalian cells, suggests enhanced local lipid synthesis that contributes to phagophore growth directly or indirectly by establishing a gradient in lipid composition. C. The phagophore–ER contact is captured in situ by cryo-ET. Densities spanning between the two organelles (white arrow) in an area of close juxtaposition potentially correspond to lipid transfer proteins, such as Atg2 or Vps13. Interestingly, in this example, the ER tubule seems to also be in close contact with a lipid droplet. Scale bar 200 nm. (Adapted from Bieber et al., 2022.) D. The elusive role of lipid droplets in autophagy. These organelles have been shown to form direct contacts with phagophores (Figure 1), and may contribute lipids both directly or indirectly by replenishing the ER.

While the vacuolar localization of the PAS appears to be a specific characteristic of yeast autophagy, interactions of the phagophore with the ER have been robustly observed in both mammalian cells (Axe et al., 2008; Hayashi-Nishino et al., 2009; Ylä-Anttila et al., 2009) and yeast (Graef et al., 2013; Suzuki et al., 2013). In mammalian cells, the omegasome, an ER subdomain transiently enriched for PI3P and the PI3P-binding protein DFCP1, has been suggested to act as a platform for autophagosome biogenesis (Axe et al., 2008; Karanasios et al., 2013). In EM data of starved mammalian cells, the ER is often observed to cradle the phagophore (Hayashi-Nishino et al., 2009), usually covering around 20% of the phagophore but sometimes enwrapping half of it or more (Takahashi et al., 2022). While such ER cradles have not been observed in yeast, it is noteworthy that these extended ER contacts in mammalian cells often include the phagophore rim, where the vast majority of phagophore–ER contacts are observed in yeast (Bieber et al., 2022; Graef et al., 2013; Suzuki et al., 2013). Regarding the type of ER, most phagophores were observed by cryo-ET to contact cytoplasmic ER tubules in yeast (Figure 3); however, in around one out of five cases, the contact was established with the nuclear membrane instead (Figure 1) (Bieber et al., 2022).

### Lipid Transfer Proteins and Scramblases are Located at the Phagophore-ER MCS

From the combined work of different groups, the emerging model is that these ER–rim contacts are the sites of lipid transfer from the ER to the phagophore which ultimately allow the early phagophore to expand into a fully-grown autophagosome within ten minutes from start to finish (Figure 3A and B) (Geng et al., 2008; Schütter et al., 2020; Tsuboyama et al., 2016). The key protein for membrane expansion is Atg2, a protein that was shown to tether membranes and shuttle lipids between them in vitro, presumably through a hydrophobic groove (Maeda et al., 2019; Osawa et al., 2019, 2022; Valverde et al., 2019). Deletion of Atg2 or its interactors inhibits autophagy (Suzuki et al., 2007; Tsukada and Ohsumi, 1993) and mutations disrupting its lipid transfer capability have been shown to affect autophagy in vivo (Osawa et al., 2019; Tan and Finkel, 2022; Valverde et al., 2019). Both Atg2 in yeast and its mammalian homolog ATG2A localize to the phagophore–ER contact site in vivo (Graef et al., 2013; Suzuki et al., 2013; Valverde et al., 2019). Specific binding of the Atg2 C-terminus to the phagophore membrane is likely mediated by interactions with PI3P (via Atg18) and Atg9 (Gómez-Sánchez et al., 2018; Obara et al., 2008b; van Vliet et al., 2022). While the N-terminus of Atg2 was shown to be capable of lipid extraction from membranes, it is currently unclear how it gets directed specifically to the ER or ERES (Osawa et al., 2019). In addition to Atg2, it was suggested that another protein of the same family, Vps13, likewise participates in lipid transfer from the ER into the phagophore (Dabrowski et al., 2022). For either of these transfer proteins, lipid channeling is thought to occur initially from the cytoplasmic leaflet of the ER membrane to the cytoplasmic leaflet of the phagophore membrane. The only transmembrane protein in the core autophagy machinery, Atg9, is a lipid scramblase, capable of redistributing lipids between membrane leaflets of the phagophore membrane in an ATP-independent manner (Maeda et al., 2020; Matoba et al., 2020). In mammalian cells, the lipid scramblases TMEM41B and VMP1 fulfill this function on the ER side (Ghanbarpour et al., 2021; Huang et al., 2021; Li et al., 2021). In yeast, however, members of this protein family are nonessential for autophagy, and no alternative proteins have been identified yet (Osawa et al., 2022). For a more in-depth discussion on Atg2 and Atg9-mediated lipid transport, the reader is referred to comprehensive reviews on this topic (McEwan and Ryan, 2022; Noda, 2021; Osawa et al., 2022).

### Lipid Transfer Kinetics at the Phagophore-ER MCS

The predominant function of phagophore–ER MCSs appears to be lipid transfer. Autophagosomes observed by cryo-ET have a tight intermembrane distance (around 8.9 nm) and a much higher ratio of membrane area to intermembrane lumen than typical vesicles in the cell. Therefore, vesicle fusion is thought to contribute no more than 40% of the membrane, which means that 60% or more of the autophagosome phospholipids are likely delivered via lipid transfer (Bieber et al., 2022). What are the lipid transfer kinetics of Atg2 and how many copies of this protein are needed at the MCS? The yeast phagophore rim is not lined completely with ER and only few densities potentially corresponding to lipid transfer proteins were observed between the two organelles, suggesting that these proteins may be present in relatively low copy numbers (Bieber et al., 2022). To measure the number of Atg2 molecules per phagophore, Dabrowski, Tulli and Graef applied quantitative live-cell fluorescence microscopy to yeast cells expressing Atg2-3GFP. They measured a maximum of 64 ± 23 Atg2 molecules per phagophore during expansion and estimated an *in vivo* lipid transfer rate of around 200 phospholipids/second/Atg2. This rate is in line with previous rates determined by FRET-based lipid transfer assays (Maeda et al., 2019; Osawa et al., 2019; Von Bülow and Hummer, 2020). In wild-type yeast, lipid transfer does not seem to limit the rate of autophagosome biogenesis, which is explained by the presence of Vps13 acting as a second lipid transporter in parallel to Atg2 (Dabrowski et al., 2022).

### Several Factors may Drive Lipid Transfer from the ER to the Phagophore

How is unidirectional lipid transport from the ER to the phagophore ensured? In other words, where does the difference in free energy come from, given that neither Atg2 nor the lipid scramblases are ATPases? One compelling theory is that local lipid synthesis in the cytoplasmic leaflet of the ER close to Atg2 generates a difference in chemical potential necessary for driving lipid transfer (Melia and Reinisch, 2022; Osawa et al., 2022). Several lines of evidence support this theory: First, phosphatidylinositol synthase was found to be enriched in the ER close to autophagosome formation sites in mammalian cells (Nishimura et al., 2017). Second, local lipid synthesis involving the Acyl-CoA synthetase Faa1 and its downstream machinery has been shown to drive phagophore expansion (Schütter et al., 2020). Third, newly synthesized phosphatidylcholine is enriched in autophagosomes, distributed equally to both leaflets in an Atg9-dependent manner (Ogasawara et al., 2020a; Orii et al., 2021). Lastly, the Kennedy pathway enzyme CCTβ3 was found to sustain autophagy after prolonged starvation of mouse embryonic fibroblasts (Ogasawara et al., 2020a). In addition to lipid synthesis, crowding of proteins like lipidated Atg8 in the phagophore membrane and lipid packing defects due to the high curvature at the phagophore rim were suggested to drive flux (Melia and Reinisch, 2022; Zhang et al., 2022). Notably, in vitro lipid transfer activity by Atg2 is stronger for small liposomes (≤80 nm) than for larger ones (≥140 nm) (Osawa et al., 2019). Complementary to in vitro assays, in situ cryo-ET allows accurate measurements of membrane morphology and curvature at native contact sites (Figure 3C). It is noteworthy that at putative Atg2 densities in the cryo-tomograms of yeast phagophores, the local membrane curvedness was on average 0.10 nm^−1^ at the phagophore vs 0.02 nm^−1^ at the ER or nuclear membrane (Bieber et al., 2022). For reference, assuming a local membrane geometry similar to a half cylinder, this corresponds to radii of around 7 nm (phagophore) vs >30 nm (ER). This indicates not only that the membrane curvatures in play are likely much higher than those previously assayed in vitro (Osawa et al., 2019) but also that there might be a local curvature gradient between the donor and acceptor membrane which could affect lipid transfer. For example, similarly to how lipid synthesis restricted to one leaflet would cause a difference in membrane tension between the leaflets of the ER membrane, a membrane tension gradient could likewise be established at the phagophore rim by the high local curvature (Lipowsky, 2022). On the other hand, how the presence of scramblases affects such gradients remains to be determined since they might at least partially counteract them by facilitating the equilibration of lipids between the leaflets (Osawa et al., 2022).

All in all, our understanding of lipid transfer from the ER to the phagophore as an essential process for phagophore expansion has advanced in great steps in the past few years. Next, to identify potential yet unknown players in this process, it would be useful to obtain close-to-native counts and kinetics of the known players. Precise in situ measurements of lipid composition (Schmitt et al., 2022; Schütter et al., 2020) and membrane curvatures (Bieber et al., 2022) can serve as a basis to design *in vitro* assays mimicking the native situation even more closely, thus yielding more reliable estimations, e.g. of transfer kinetics. Going forward, one would wish for a model integrating all the pieces of information obtained from different experiments—membrane curvature, lipid composition, local lipid synthesis, and protein crowding as well as lipid transfer and interleaflet scrambling kinetics—for a more complete understanding of phagophore expansion.

## Phagophore-LD Interactions

Lipid droplets (LDs) are essential for starvation-induced autophagy, which is inhibited in the absence of LDs (Shpilka et al., 2015). One explanation for this is indirect contribution, e.g. by sustaining ER homeostasis, thus supporting continued lipid transfer from the ER into growing phagophores (Velázquez et al., 2016). Interestingly, several proteins have been linked both to autophagy and lipid droplet homeostasis, tightening the link between autophagy and lipid metabolism (Ogasawara et al., 2020b). A prominent example is the lipid transfer protein ATG2A which has been shown to localize to LDs in human cell lines and plays a role in lipid homeostasis by regulating the size of LDs (Nishimura et al., 2017; Pfisterer et al., 2014; Velikkakath et al., 2012). The scramblases Atg9, TMEM41B, and VMP1 have also been shown to play a dual role in LD metabolism and autophagy (Mailler et al., 2021; Ogasawara et al., 2020b). While most authors tend to agree that these proteins may link autophagosome and LDs through their common interactor, the ER (Mailler et al., 2021; Ogasawara et al., 2020b), it is also possible that LDs might interact directly with phagophores to provide lipids (Figure 3D). LDs were in fact shown to transiently contact the growing phagophore both in yeast and mammalian cells (Dupont et al., 2014; Li et al., 2015; Shpilka et al., 2015). In MEFs, LDs were not observed within 30 nm of phagophores after short starvation times (30 min) (Takahashi et al., 2022); however, during prolonged starvation (8 h), phagophores appear to emerge from LD-rich areas, concomitant with CCTβ3 localizing to LDs (Ogasawara et al., 2020a). Upon overexpression of CCTβ3, EM data revealed putative phagophores contacting LDs with their rim (Ogasawara et al., 2020a). Strikingly, in cryo-ET data of nitrogen-starved yeast cells (starvation time 0.5–3 h), a small number of phagophores were likewise observed to contact LDs with the rim, evident from the deformations of the rim towards the LD (Bieber et al., 2022). We can thus speculate that during autophagy, LDs are not only essential for ER homeostasis but also could act as optional direct lipid sources for phagophore expansion. The importance of this second mechanism likely varies depending on the cellular state, gaining importance, for example, upon prolonged starvation (Ogasawara et al., 2020a). It will be interesting to explore these phagophore–LD interactions in different cell types and stimuli in the future.

## Concluding Remarks

From the first electron micrographs of phagophores amid other organelles in fixed cells, our understanding of the structure, scope, and function of MCSs in autophagy has by now advanced tremendously due to the integration of biochemical, imaging, and structural studies. In addition to the contact sites discussed here, going forward, it will be interesting to explore how metabolic conditions and various types of cargo influence the established contact sites and their functional role (Zwilling and Reggiori, 2022). Systematic screenings involving many cell types and conditions might help to reveal the underlying principles and mechanisms explaining the variable involvement of different organelles in autophagosome biogenesis. Furthermore, with the streamlining and increasing availability of cryo-CLEM, using this method to characterize MCSs in mammalian autophagy holds great potential to help decipher their structure and function. Such studies can also contribute to our understanding of the degree of conservation of autophagy from yeast to mammals, potentially revealing important similarities and differences beyond the ones known from the protein machinery.
